# The amino terminus of PetD is essential for cytochrome *b*_6_ *f* function and the negative feedback control of STT7 kinase

**DOI:** 10.1038/s41477-026-02310-y

**Published:** 2026-06-02

**Authors:** Afifa Zaeem, Yuval Milrad, Simon Bütfering, Carolyne Stoffel, Sandrine Bujaldon, Adrien Burlacot, Martin Scholz, Felix Buchert, Michael Hippler

**Affiliations:** 1 Institute of Plant Biology and Biotechnology, University of Münster, Münster, Germany; 2 Biosphere Sciences and Engineering Division, Carnegie Science, Stanford, CA USA; 3 Laboratoire de Photobiologie et Physiologie des Plastes et des Microalgue, UMR7141, Institut de Biologie Physico-Chimique, CNRS / Sorbonne Université, Paris, France; 4 Department of Biology, Stanford University, Stanford, CA USA; 5 Institute of Plant Science and Resources, Okayama University, Kurashiki, Japan

**Keywords:** Light stress, Non-photochemical quenching

## Abstract

The cytochrome *b*_6_ *f* complex (*b*_6_ *f*) links photosystem (PS) I and PSII in the photosynthetic electron transport chain and is distributed across appressed and non-appressed thylakoid membranes. The *b*_6_ *f* also activates the state-transition 7 kinase (STT7), which phosphorylates light-harvesting complex proteins, triggering their migration to enable energy redistribution between PSII and PSI. STT7-dependent phosphorylation has also been observed at Thr4 in the amino-terminal domain of the *b*_6_ *f* subunit-IV (PetD), though its functional significance has remained unclear. Here, to investigate its role, we generated several chloroplast mutants. The phosphomimic substitution PetD T4E—but not T4A—inhibited STT7 kinase activity, as indicated by the absence of STT7-dependent phosphorylation and a State 1-locked phenotype. This reveals a feedback mechanism regulating STT7-dependent phosphorylation. The deletion of the first five N-terminal amino acids similarly inhibited STT7 activity and additionally disrupted electron transfer, underscoring a crucial role of the PetD N terminus in *b*_6_ *f* function.

## Abstract (web summary)

This study reveals that the cytochrome *b*_6_*f* subunit PetD regulates the STT7 kinase. In particular, the N terminus is required for kinase activation, while Thr4 phosphorylation provides feedback that impairs kinase activity and state transitions.

## Main

Photosynthesis captures light through photosystem (PS) I and PSII along with light-harvesting complexes (LHCI and LHCII). Light energy drives water splitting at PSII and ultimately reduces ferredoxin (FDX) through PSI. In linear electron flow, reduced FDX transfers electrons to FDX–NADP(H) oxidoreductase, producing NADPH for CO_2_ fixation and biomass production. In addition, electron transport deposits H^+^ in the lumen and separates charges across the thylakoid membrane, thereby generating both a proton gradient and a membrane potential, which collectively drive ATP synthesis. Electron flow between the photosystems is mediated by the cytochrome *b*_6_ *f* complex (*b*_6_ *f*; reviewed in ref. ^1^), which shuttles electrons from plastoquinol (PQH_2_) in the plastoquinone (PQ) pool to plastocyanin. Besides small peripheral subunits, the *b*_6_ *f* core comprises cytochrome *f* (PetA), cytochrome *b*_6_ (PetB), Rieske iron–sulfur protein (PETC) and subunit-IV (PetD). Electron flow through the high-potential chain, involving PETC and PetA, is functionally linked to redox reactions in the low-potential chain of PetB, including the haeme *c*_i_ cofactor at the Q_i_ site. The Q_i_ site serves as the stromal binding pocket for PQ, whereas PQH_2_ binds at the lumenal Q_o_ site. By translocating protons into the lumen through reversible PQ protonation at the Q_i_ and Q_o_ sites, the proton gradient generated by *b*_6_ *f* activity substantially contributes to ATP production required for CO_2_ fixation. Moreover, the *c*_i_ haeme in the Q_i_ site is relevant for cyclic electron flow (CEF), and the *b*_6_ *f* complex may function as an FDX-PQ reductase in CEF^2,3^.

**Extended Data Fig. 1:**
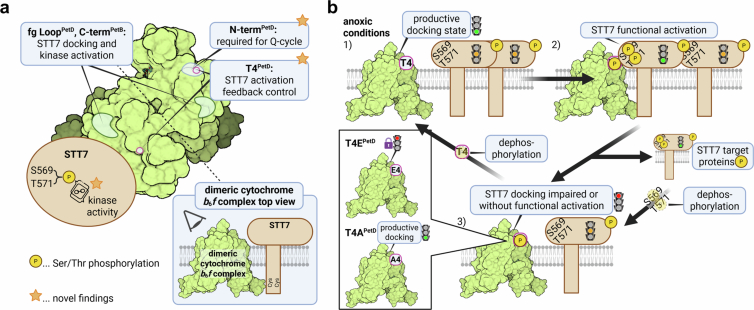
Graphical abstract of the cytochrome *b*_6_*f* complex interplay with STT7 kinase regulation. **a**, The N-terminus of PetD has been investigated and the focus was on T4. The model is built on previous STT7 docking studies (refs. ^17,18^ in the main text) and disregards possible STT7 oligomerization and redox regulation for the sake of simplicity. **b**, The feedback loop upon PetD T4 phosphorylation as well as investigated mutants is summarized. Figure created in BioRender; Buchert, F. https://biorender.com/8gai3b7 (2026).

**Extended Data Fig. 2:**
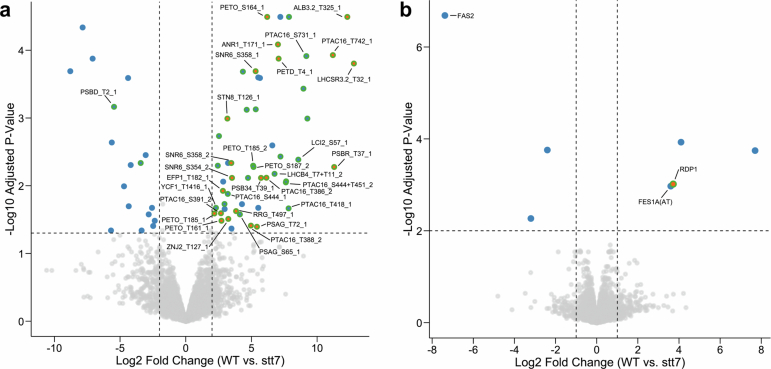
Volcano plots visualizing differential phosphorylation (a) and protein expression (b) between WT and *STT7* mutant. Differential expression analysis was performed using the ‘limma’ package implemented in Phospho-Analyst. P-values were corrected for multiple testing using the Benjamini-Hochberg method. Proteins with adjusted p-value < 0.01 and |log2 fold change |> 1, and phosphorylations with adjusted p-value < 0.05 and |log2 fold change | > 2 are highlighted in blue, with threshold boundaries shown as dashed lines. Data points are shown in orange instead of blue when all values for one strain in the fold-change calculation are imputed due to completely missing data. Green halos indicate chloroplast-encoded proteins and nuclear-encoded proteins with confirmed or predicted (PB-Chlamy) chloroplast localization.

The lumen acidification stemming from photosynthetic electron flows is important for efficient photoprotection under conditions of excess excitation energy: it establishes photosynthetic control at the Q_o_ site, where PQH_2_ oxidation (and therefore electron flow) slows down at acidic pH^4,5^, and it decreases photochemistry by stimulating controlled heat dissipation via non-photochemical quenching^6–8^. Moreover, *b*_6_ *f* is linked to state transitions—another photoprotective mechanism to cope with imbalanced excitation^9^. During this process, LHCII migrates between PSII and PSI upon its phosphorylation by the thylakoid-bound state-transition 7 kinase (STT7)^10,11^. STT7 is activated by a reduced PQ pool, while specific phosphatases act as STT7 antagonists^12^. The activity of the STT7 kinase is regulated by its transmembrane domain, with a disulfide bond between two lumenal cysteine residues being essential for its function^13^. Active discussions and research on the kinase redox regulation are ongoing^14–16^. It was recently shown that state transitions involve PetD residues Asn122, Tyr124 and Arg125 in the stromal fg loop, linking helices F and G of this *b*_6_ *f* subunit^17^. Moreover, the carboxy terminus of the *b*_6_ *f* subunit PetB (PetB^C-term^) interacts with the fg loop in PetD, thereby facilitating STT7 activation^18^. Mechanistically, the fg loop and PetB^C-term^ allow for the binding of STT7 to *b*_6_ *f*, which enhances kinase autophosphorylation. In this Letter, we focus on another stromal element of *b*_6_ *f* in the model green microalgae *Chlamydomonas reinhardtii* (Extended Data Fig. 1): the amino terminus in PetD, where Thr4 undergoes phosphorylation in an STT7-dependent manner^19^ (see also Extended Data Fig. 2).

**Extended Data Fig. 3:**
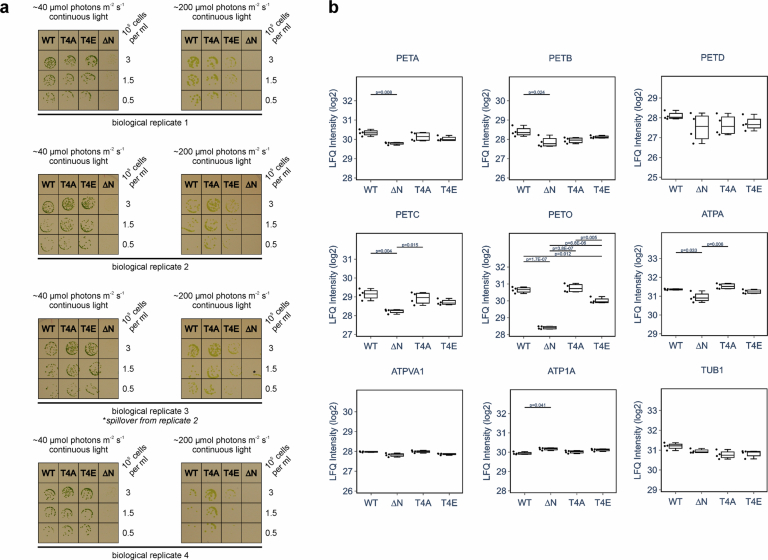
Growth analysis in varying light conditions and protein expression in the mutants. **a**. Spot tests on minimal media compare the growth under two light conditions (40 and 200 µmol photons m^−2^ s^−1^). **b**. Protein expression of *b*_6_ *f* subunits and control proteins in the engineered PetD strains. Boxes span the interquartile range (IQR) with the median shown as a horizontal line. Whiskers extend to the most extreme data points within 1.5× IQR. The individual data points of the four biological replicates (*n* = 4) are overlaid as dots. Differential expression analysis was performed using the ‘limma’ package implemented in Phospho-Analyst, utilizing linear models in combination with empirical Bayes statistics. If the dataset has three or more experimental groups, an ANOVA test is performed in addition to moderated t-tests. P-values were corrected for multiple testing using the Benjamini-Hochberg method. Horizontal lines indicate statistically significant pairwise comparisons between strains.

**Extended Data Fig. 4:**
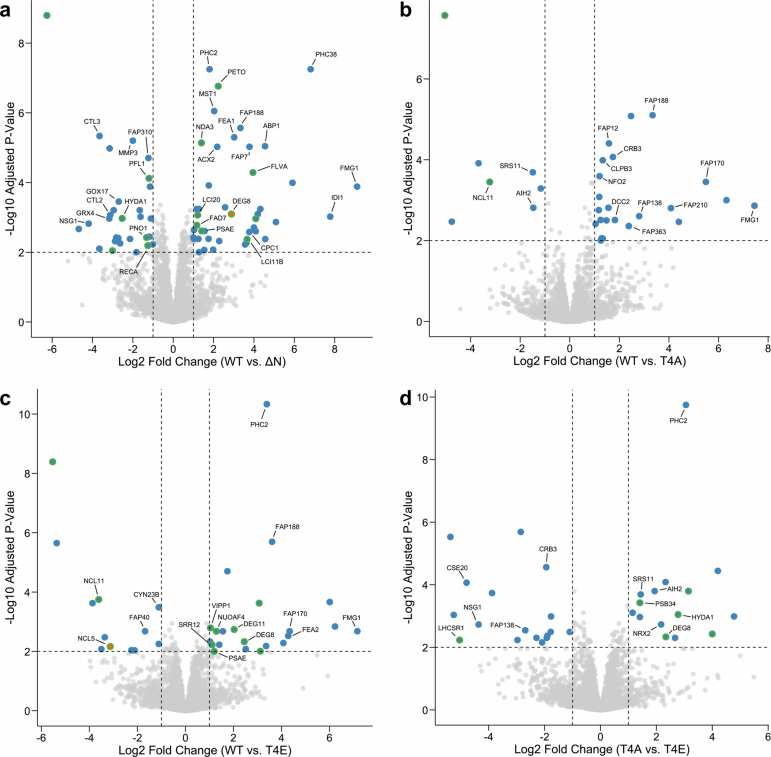
Volcano plots visualizing differential protein expression between WT and PetD mutants. Differential expression analysis was performed using the ‘limma’ package implemented in Phospho-Analyst, utilizing linear models in combination with empirical Bayes statistics. If the dataset has three or more experimental groups, an ANOVA test is performed in addition to moderated t-tests. P-values were corrected for multiple testing using the Benjamini-Hochberg method. Proteins from four biological replicates (*n* = 4) with adjusted p-value < 0.01 and |log_2_ fold change| > 1 are highlighted in blue, with threshold boundaries shown as dashed lines. Data points are shown in orange instead of blue when all values for one strain in the fold-change calculation are imputed due to completely missing data. Green halos indicate chloroplast-encoded proteins and nuclear-encoded proteins with confirmed or predicted (PB-Chlamy) chloroplast localization. Comparisons are shown for **a** WT vs. ΔN, **b** WT vs. T4A, **c** WT vs. T4E, and **d** T4A vs. T4E.

**Extended Data Fig. 5:**
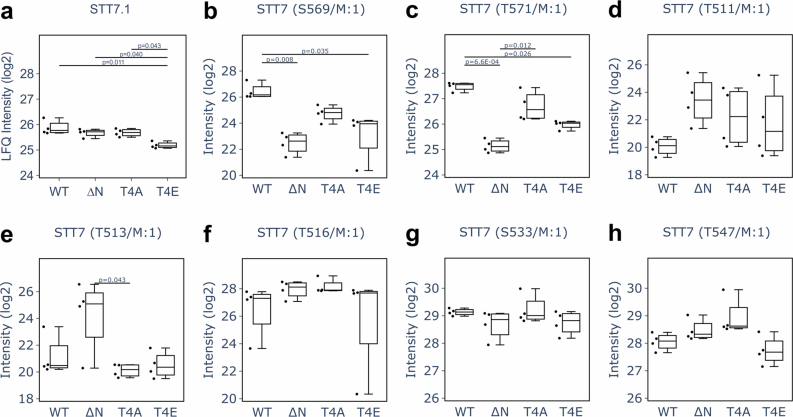
STT7 protein expression and selected autophosphorylation amounts in the engineered PetD strains. The box plots visualize significant differences of log_2_ fold changes in (**a**) protein LFQ and (**b**, **c**) phosphosite intensities from four biological replicates. No significant intensity differences were detectable for various STT7 phospho-peptides (panels **d** – **h**). Boxes span the interquartile range (IQR) with the median shown as a horizontal line. Whiskers extend to the most extreme data points within 1.5× IQR. The individual data points of the four biological replicates (*n* = 4) are overlaid as dots. Differential expression analysis was performed using the ‘limma’ package implemented in Phospho-Analyst, utilizing linear models in combination with empirical Bayes statistics. If the dataset has three or more experimental groups, an ANOVA test is performed in addition to moderated t-tests. P-values were corrected for multiple testing using the Benjamini-Hochberg method. Horizontal lines indicate statistically significant pairwise comparisons between strains.

PetD Thr4 phosphorylation has been described by several groups^19,20^. To investigate the functional significance of STT7-dependent phosphorylation at PetD Thr4, we created PetD site-directed mutants T4A and T4E via chloroplast transformation to mimic the constitutive absence and presence of phosphorylation, respectively. Moreover, the function of the five N-terminal residues was addressed in a truncation mutant (ΔN), whereas the wild-type (WT) PetD was used as the control. The *petD* recipient strain carried a six-histidine tag at the carboxy terminus of PetA^21^ for optional *b*_6_ *f* purification. Spot tests on agar plates, requiring photoautotrophic growth, revealed that mutant strains T4A and T4E performed similarly to the WT under normal light (40 μmol photons m^−2^ s^−1^; Extended Data Fig. 3a). In moderately high light (200 μmol photons m^−2^ s^−1^; Extended Data Fig. 3a), T4E revealed a severe growth phenotype in comparison to T4A and the WT. In contrast, the ΔN strain showed a growth deficit at both light intensities compared with the other strains. Proteomic analyses showed no significant differences in the levels of PetD and other core *b*_6_ *f* subunits between the WT, T4A and T4E. In contrast, most of these subunits were slightly depleted in ΔN, and the CEF effector protein PETO^22^ was significantly downregulated (Extended Data Fig. 3b). LHCSR1—required for pH-dependent non-photochemical quenching^23^—was markedly upregulated in T4E relative to T4A (Extended Data Fig. 4). Moreover, STT7 peptide abundance was slightly lower in T4E than the similar levels observed in the other PetD variants (Extended Data Fig. 5a). Given the observed growth and protein abundance phenotypes, key photoprotection pathways associated with *b*_6_ *f* function might be altered in the mutants. To explore this possibility, we first investigated state transitions, as they require the physical presence of *b*_6_ *f* (ref. ^24^), and then performed electron transfer measurements to directly assess *b*_6_ *f* activity in the mutants.

**Fig. 1:**
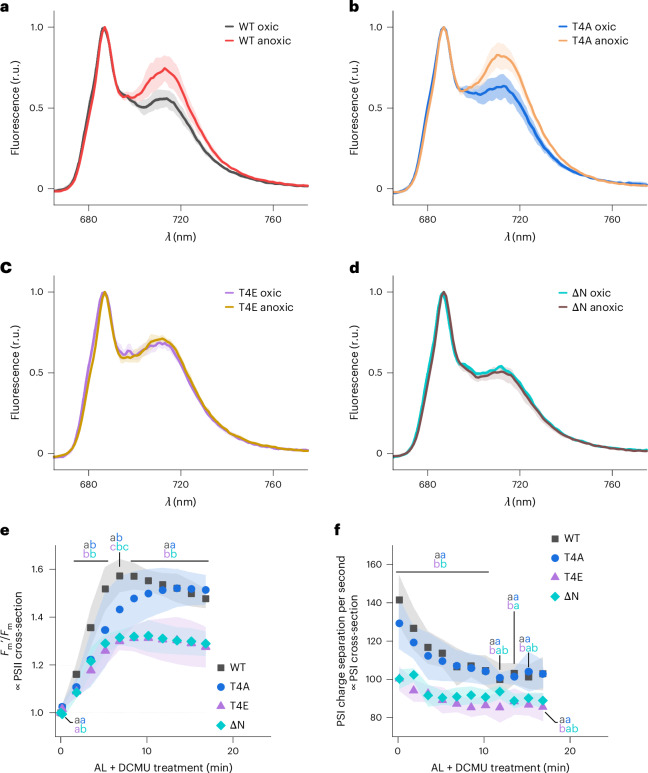
Assessment of relative antenna cross-section by measuring chlorophyll fluorescence and electrochromism. **a**–**d**, The differences in antenna attached to PSI under State 1 (oxic) and State 2 (anoxic), contributing to emission signals at a wavelength (*λ*) of ~712 nm. The 77 K emission spectra are expressed in relative units (r.u.) by normalizing to PSII-attached antenna signals at ~687 nm after baseline correction. The data are presented as mean values ± s.d. (*n* = 3 biological replicates). **e**, The rise in *F*_m_′, normalized to *F*_m_, was recorded in DCMU-treated anaerobic cells during light-driven PQ pool oxidation. The data are presented as mean values ± s.d. (*n* = 4 biological replicates). The letters indicate statistical significance determined by one-way analysis of variance (ANOVA) with Fisher’s least significant difference post hoc test (*P* < 0.05). AL, actinic light. **f**, Under the same conditions as in **e**, the initial slope of ECS signals was recorded during short light pulses and normalized to PSI. The data are presented as mean values ± s.d. (*n* = 4 biological replicates). The letters indicate statistical significance determined by one-way ANOVA with Fisher’s least significant difference post hoc test (*P* < 0.05). Source data

To explore whether state transitions are affected in the mutants, we measured the antenna redistribution between PSI and PSII using 77 K fluorescence emission spectroscopy^25^ after cells were shifted to either State 1- or State 2-inducing conditions (Fig. 1a–d). An increase in PSI-associated antennae size under State 2 versus State 1 conditions was thereby observed for the WT and T4A (Fig. 1a,b). In contrast, T4E and ΔN strains displayed no differences in their 77 K fluorescence emission spectra, indicating that they were unable to perform state transitions under these conditions (Fig. 1c,d). We also monitored the transition from State 2 to State 1 at ambient temperature under continuous illumination while PSII was inhibited (Fig. 1e,f). The rise in maximal chlorophyll fluorescence during illumination (*F*_m_′) indicated an increase in PSII antenna size (Fig. 1e). This response was most effectively produced in the WT (~50% higher fluorescence yield) and in T4A, though with a delay. In contrast, the increase in *F*_m_′ was about half as large in T4E and ΔN. Conversely, electrochromic shift (ECS) measurements^26^ revealed a progressive decrease in PSI antenna size (Fig. 1f). This effect occurred in the WT (~27% reduction from 141 to 103 PSI charge separations per second) and in T4A, whereas T4E and ΔN showed a pronounced impairment (Fig. 1f). The amplitudes of fluorescence- and ECS-based state transitions in the WT were consistent with values reported in the literature^27^.

**Fig. 2:**
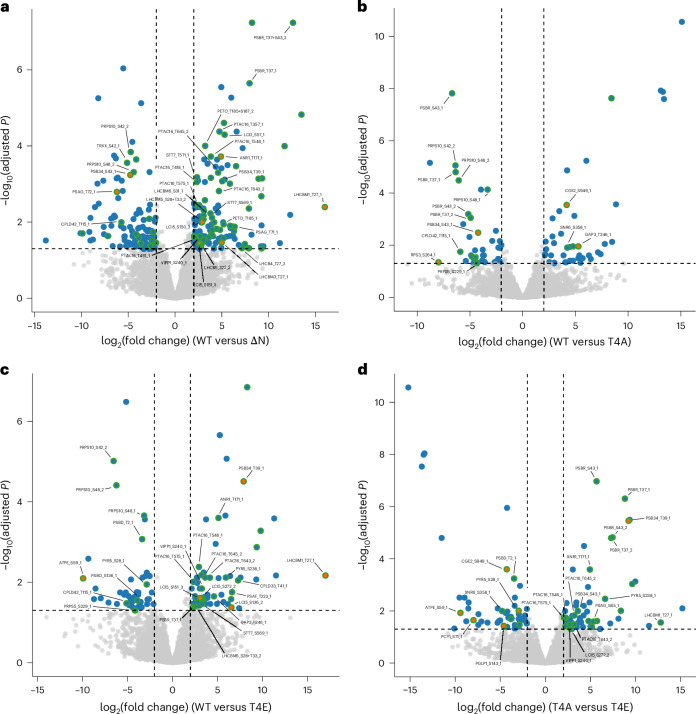
Volcano plots visualizing the effect of PetD alterations on protein phosphorylation. **a**–**d**, Differential expression analysis was performed using the limma package implemented in Phospho-Analyst. *P* values were corrected for multiple testing using the Benjamini–Hochberg method. Phosphorylation sites from four biological replicates (*n* = 4) with adjusted *P* < 0.05 and |log_2_(fold change)| > 2 are highlighted in blue, with threshold boundaries shown as dashed lines. Data points are shown in orange instead of blue when all values for one strain in the fold-change calculation were imputed due to completely missing data. Green halos indicate chloroplast-encoded proteins and nuclear-encoded proteins with confirmed or predicted (PB-Chlamy) chloroplast localization. Phosphorylation log_2_ fold changes are normalized to the corresponding protein abundance levels to account for changes in protein expression. Numbers at the ends of data labels indicate peptide multiplicity (total phosphorylations per peptide; for example, PRPS10_S42_2 indicates PRPS10 with phosphorylated Ser42 and one additional phosphorylation on the same peptide). Comparisons are shown for WT versus ΔN (**a**), WT versus T4A (**b**), WT versus T4E (**c**) and T4A versus T4E (**d**).

**Extended Data Fig. 6:**
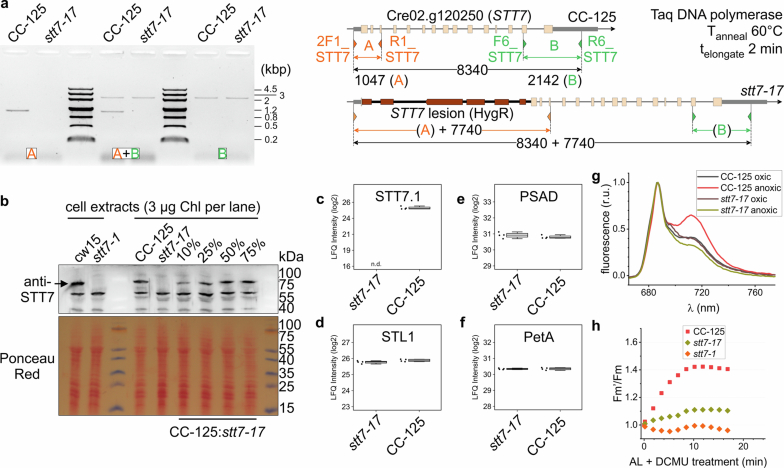
Generation and characterization of the *stt7-17* CRISPR/Cas mutant. **a**. Indirect PCR-assisted visualization of the insertion locus using genomic DNA of the *stt7-17* mutant and its control (CC-125). The map of the STT7 gene showing the hygromycin resistance cassette and fragments of it, as well as the primer binding sites are visualized (see Supplementary Table 1 for *stt7-17* genomic sequence and used primers). **b**. Compared to their respective control strains, immunodetection analyses show absence of STT7 signals (arrow) in *stt7-17* and an independent *stt7-1* mutant (main text ref. ^10^). Box plots in panels **c** to **f** visualize the log_2_ protein LFQ intensities of STT7 (n.d. not detected), STL1, PSAD, and PetA, respectively. Boxes span the interquartile range (IQR) with the median shown as a horizontal line. Whiskers extend to the most extreme data points within 1.5× IQR. Individual data points of four biological replicates (*n* = 4) are overlaid as dots. Differential expression analysis was performed using the ‘limma’ package implemented in Phospho-Analyst, utilizing linear models in combination with empirical Bayes statistics. If the dataset has three or more experimental groups, an ANOVA test is performed in addition to moderated t-tests. P-values were corrected for multiple testing using the Benjamini-Hochberg method. **g**. 77 K chlorophyll fluorescence emission spectra were carried out as in Fig. 1 of the main text, using *stt7-17* and its control CC-125. **h**. Chlorophyll fluorescence-based state transition kinetics measurements were carried out as in Fig. 1 of the main text, using *stt7-17* and its control CC-125. Moreover, an independent *stt7-1* mutant (main text ref. ^10^) was included.

The data in Fig. 1 indicate a clear defect in state transitions in T4E and ΔN and suggest impaired STT7-dependent phosphorylation in both T4E and ΔN strains. To define specific STT7-dependent phosphorylation targets, we conducted phosphoproteomic analyses including a CRISPR–Cas9-generated *stt7-17* knockout mutant (Extended Data Fig. 6). This *stt7-17* mutant served both as a benchmark reference for the PetD strains and as a tool to distinguish between effects stemming from different genetic *stt7* backgrounds (see also Supplementary Table 1). To dissect STT7 function (Fig. 2 and Extended Data Fig. 2), the following selection of results is loosely categorized into five groups: (1) kinase autophosphorylation, (2) LHCII and state transition complex formation, (3) photoprotective CEF and non-photochemical quenching, (4) *b*_6_ *f* and photosystem core subunits and (5) miscellaneous phosphorylation targets.

An unexpected^19^ observation was that, unlike *stt7-17*, the ΔN and T4E strains showed no detectable changes in LHCSR3 (Thr32) or LHCB4 double (Thr7 + Thr11) phosphorylation. As these phosphorylation events are interrelated^28^, this suggests either residual STT7 activity in ΔN and T4E or a compensatory mechanism involving the physical presence of STT7 and possibly other chloroplast kinases such as STN8 (see the degree of functional impairments in ΔN and T4E versus *stt7-17*; Fig. 1e and Extended Data Fig. 6h). Supporting the latter hypothesis, STN8 phosphorylation at Thr126—absent in *stt7-17*—was retained in both ΔN and T4E, hinting at an intricate and potentially compensatory kinase network.

1. STT7 phosphorylation at Ser569/Thr571 was severely reduced in ΔN and T4E (Extended Data Fig. 5b,c), consistent with their failure to fully undergo state transitions. Nonetheless, STT7 autophosphorylation at Thr511, Thr513, Thr516, Ser533 and Thr547 remained largely unchanged across all strains (Extended Data Fig. 5d–h), suggesting that STT7 is partially functional in ΔN and T4E.
2. In *stt7-17*, phosphorylation was broadly lost across key threonine clusters^28^, notably the double phosphorylation in LHCB4 (Thr33 + Thr35, Thr33 + Thr37, Thr35 + Thr37) and the phosphorylation of Thr23 and Thr36 in LHCB5 (Supplementary Table 1). LHCB5 retained only minimal phosphorylation at Thr20, and LHCB4 double-phosphorylation at Thr7 + Thr11 was reduced by 6.8 log_2_-fold. Several background-independent^19,29^ phosphorylation sites absent in *stt7-17* were also either absent (Thr27 of LHCBM1) or strongly diminished (double phosphorylation of Ser28 + Thr33 in LHCBM5) in ΔN and T4E. The altered N-terminal phosphorylation in LHCBM1 and LHCBM5 probably explains the loss of state transitions in both strains, as these modifications are essential for the assembly of the PSI–LHCI–LHCII state transition complex^30^.
3. As mentioned above, *stt7-17* lacked phosphorylation in the LHCSR3 N-terminal cluster (Ser26/Ser28/Thr32/Thr33/Thr39)^28^, where up to three simultaneously phosphorylated residues were observed in the WT. Meanwhile, LHCSR3 phosphorylation showed no significant difference between the WT and the PetD variants. In contrast, ANR1 phosphorylation at Thr171 was confirmed to be a background-independent^19,29^ target of STT7. It was also undetectable in ΔN and T4E. Notably, PETO levels were reduced nearly fivefold in ΔN (Extended Data Fig. 3b). Given that PETO can chemically crosslink to Lys15 in the N-terminal region of PetD^31^, its loss in ΔN and fg-loop mutants^18^ supports a functional PetD–PETO interaction. The disruption of this interaction probably triggers PETO degradation.
4. Consistent with prior data from an independent *stt7* mutant^19^, PetD Thr4 phosphorylation was absent in *stt7-17*, while PSBD (Thr2) phosphorylation was markedly elevated (5.5 log_2_-fold). T4E exhibited a similar increase (3.4 log_2_-fold), whereas ΔN and T4A showed no significant changes at PSBD (Thr2). Both ΔN and T4E displayed reduced phosphorylation at PSB34 (Thr39), PSAG (Thr71) and PSBR (Thr37, Ser43 and Thr37 + Ser43), while ΔN specifically showed increased phosphorylation at PSAG Thr72. T4A, in contrast, exhibited a distinct profile: most phosphorylation sites were unchanged, but PSBR phosphorylation (Thr37, Ser43 and Thr37 + Ser43) was significantly elevated compared with the other strains.
5. PTAC16—a component of the plastid transcriptionally active chromosome complex and a known STN7 substrate in *Arabidopsis*^32,33^—displayed the highest number of differentially phosphorylated sites. In *stt7-17*, PTAC16 phosphorylation was largely abolished. Similar patterns were observed in ΔN and T4E, though both retained some WT-specific phosphorylation. In contrast, the T4A mutant showed no significant changes in PTAC16 phosphorylation.

**Fig. 3:**
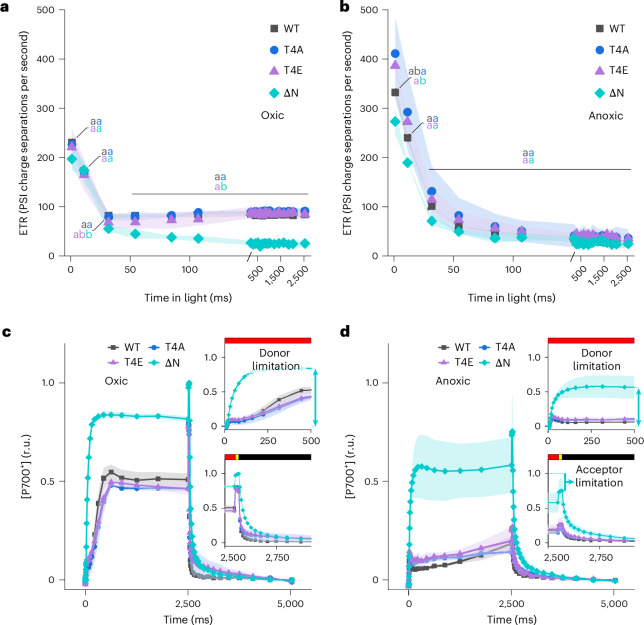
Photosynthetic activities of cytochrome *b*_6_*f* variants. **a**, ETR developed over the 2.5-s illumination period under oxic conditions, with the first detection at 1 ms of AL. The letters at the individual time points indicate statistical significance determined by one-way ANOVA with Tukey’s test (*P* < 0.05). **b**, ETR developments are shown under anoxic conditions. The letters at the individual time points indicate statistical significance determined by one-way ANOVA with Tukey’s test (*P* < 0.05). **c**, Quantification of oxidized primary PSI donor ([P700^+^]) is plotted under oxic conditions. The red, yellow and black bars represent AL intensities of 550, 3,000 and 0 µmol photons m^−2^ s^−1^. **d**, The P700 kinetics are shown as in **c** but under anoxic conditions. In all panels, averaged kinetics are presented as mean values ± s.d. (*n* = 3 biological replicates). Source data

**Extended Data Fig. 7:**
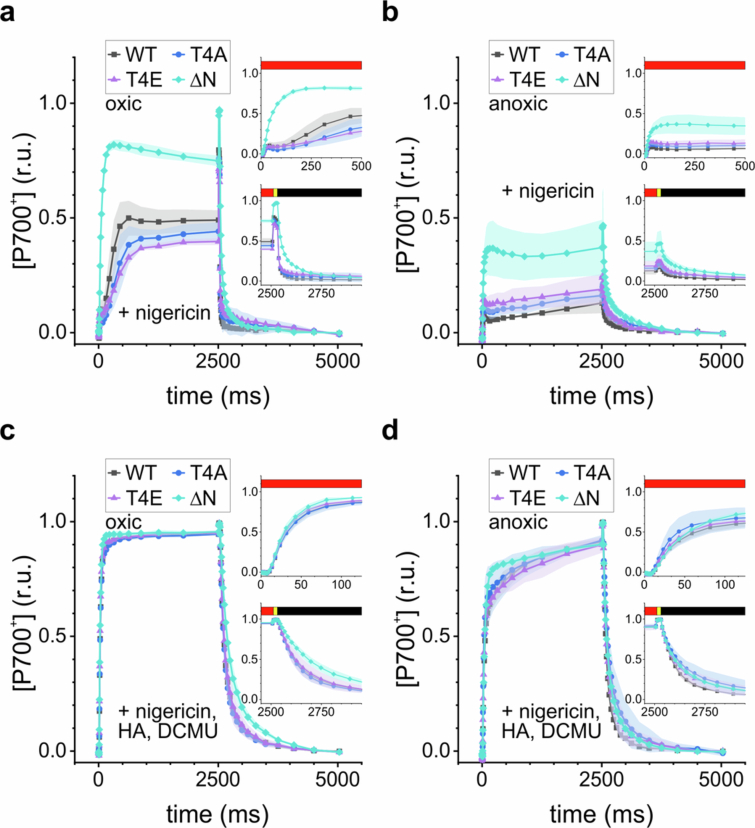
Photosynthetic activities of the cytochrome *b*_6_*f* variants for P700. Averaged kinetics are depicted (biological replicates *n* = 3 ± SD) and the reference measurements are shown in main text Fig. 3c and d. The redox changes of primary PSI donor ([P700^+^]) is plotted (**a**) under oxic and (**b**) anoxic conditions in the presence of 10 µM nigericin, acting as H^+^/K^+^ exchanger to attenuate ΔpH in favor of an electric field. Red, yellow, and black bar represent actinic light intensities of 550, 3000, and 0 µmol photons m^−2^ s^−1^. **c**, The conditions were as in panel a but photosystem II was inhibited by hydroxyl amine (HA; 1 mM) and 3-(3,4-Dichlorophenyl)-1,1-dimethylurea (DCMU; 10 µM). **d**, The conditions were as in panel c except for the anoxic pre-treatment. Source data

To assess the functional consequences of the alterations in the engineered strains, we measured photosynthetic electron transfer rates (ETR) using ECS signals. The measured ETR in T4A and T4E were indistinguishable from those in the WT, suggesting that phosphorylation at Thr4 does not have a direct impact (Fig. 3a,b). In contrast, ΔN cells showed a marked slowdown in ETR 30–50 ms after light onset under oxic conditions (Fig. 3a). Under anoxic conditions that favour CEF, ETR initially increased but reached a lower steady state, with the initial boost markedly reduced in ΔN compared with the other strains (Fig. 3b), indicating that the PetD N terminus supports ETR via *b*_6_ *f*. The redox kinetics of P700 were also assessed (Fig. 3c,d). In ΔN, P700 oxidized rapidly upon illumination and re-reduced slowly in the dark, consistent with donor-side limitation under both oxic and anoxic conditions. Notably, steady-state ETR (Fig. 3a,b) were established despite substantial P700 acceptor-side limitation in all strains except in ΔN (Fig. 3c,d). This persistent donor-side limitation in ΔN was unaffected by the addition of the H^+^/K^+^ exchanger nigericin (Extended Data Fig. 7a,b), excluding pH-dependent photosynthetic control. Treatment with 3-(3,4-dichlorophenyl)-1,1-dimethylurea (DCMU) and nigericin led to rapid P700 oxidation in all strains, slightly faster in ΔN (Extended Data Fig. 7c,d), supporting impaired *b*_6_ *f* function in both linear electron flow and CEF.

**Fig. 4:**
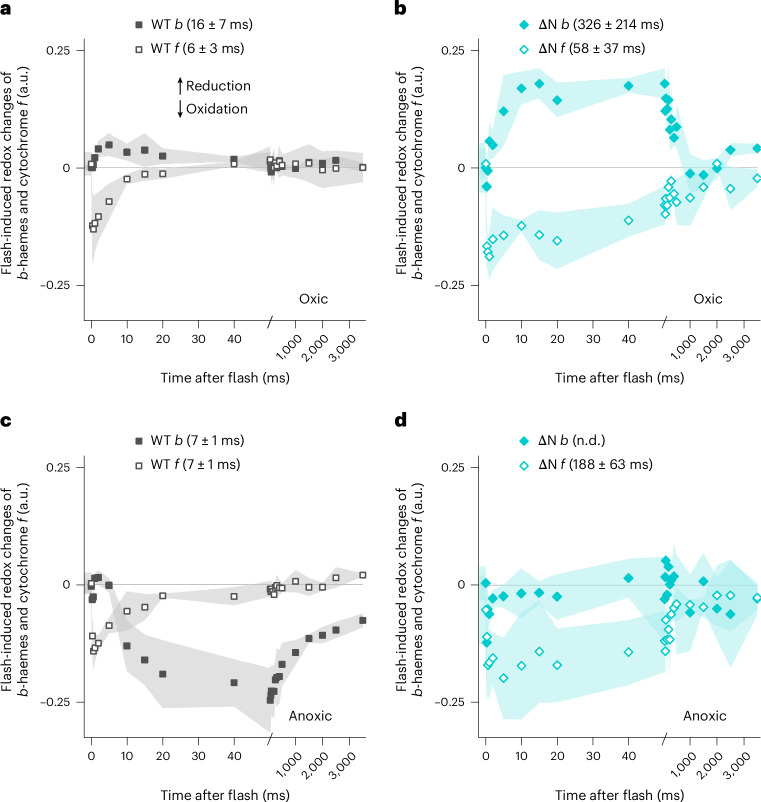
Single-turnover redox changes of cytochrome *b*_6_*f* haemes. **a**–**d**, Averaged kinetics are presented as mean values ± s.d. (*n* = 3 biological replicates). The WT (**a**,**c**) and the ΔN mutant (**b**,**d**) were measured under oxic (**a**,**b**) and anoxic conditions (**c**,**d**). The average half-times of cytochrome-*f* reduction and *b*-haeme oxidation are given in the panels’ legends (n.d., not determined). Source data

**Extended Data Fig. 8:**
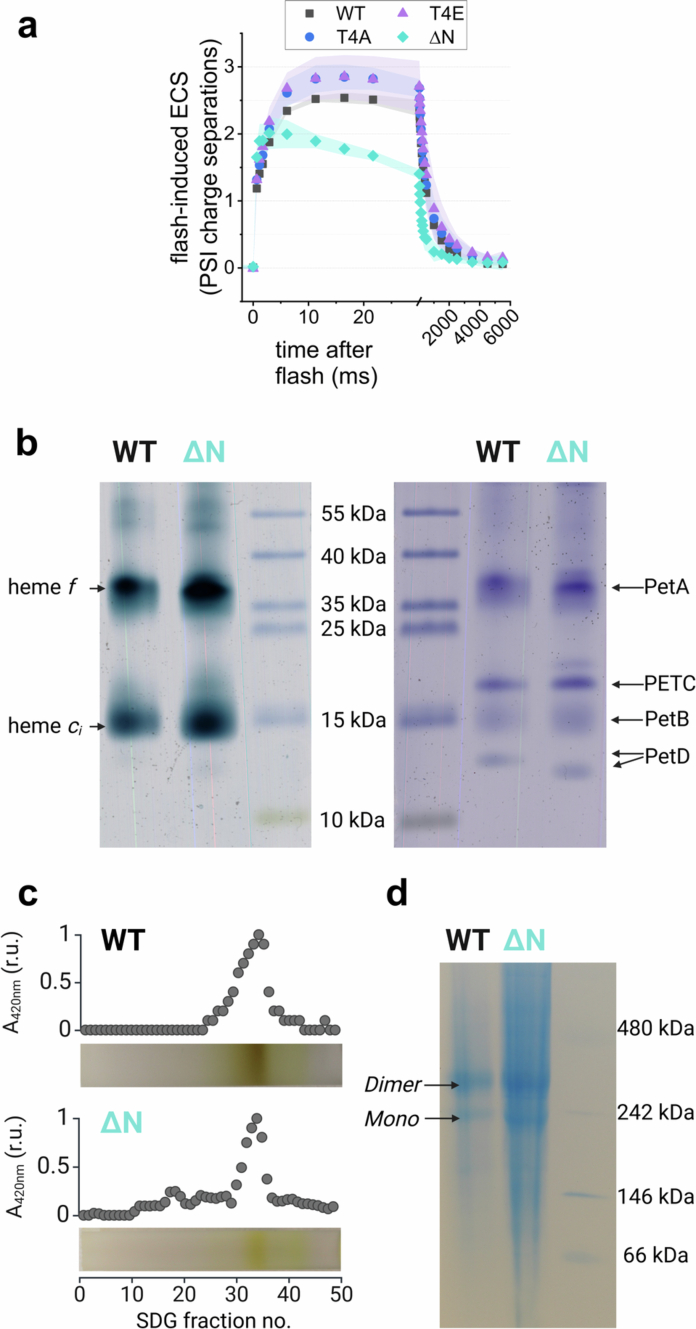
Laser flash-induced electrochromic shift (ECS) measurements show striking differences in ΔN and are not related to stability properties mutant *b*_6_*f*. **a**. The ECS shows multiphasic kinetics that rely on activities of both photosystems, the cytochrome *b*_6_ *f* complex and ATP synthase. Averaged measurements are shown (biological replicates *N* = 3 ± SD). **b**. Heme *c*_i_ was present in the ΔN mutant strain, as shown by in-gel heme staining with TMBZ. Coomassie-stained gels of 10 µg *b*_6_ *f* are shown for reference. The experiment was repeated independently at least three times with similar results. **c**. Sucrose density gradient (SDG) fractionation and **d**. BN-PAGE analysis of 5 µg pooled *b*_6_ *f* showed no increased monomerization tendency in ΔN particles. The experiment was repeated independently at least five times with similar results. Source data

To confirm whether the observed ETR and P700 redox kinetics differences stemmed from *b*_6_ *f* dysfunction, we evaluated *b*_6_ *f* electrogenicity and redox kinetics (Fig. 4 and Extended Data Fig. 8a). ECS rise linked to Q-cycle activity was impaired exclusively in ΔN, despite the presence of haeme *c*_i_—the terminal electron acceptor in the low-potential chain^34^—as well as WT-like stability of the truncated *b*_6_ *f* complex (Extended Data Fig. 8b–d). Under oxic conditions (Fig. 4a), WT *b*-haemes showed a transient reduction (~10 ms) before net oxidation (~16 ms half-life), while cytochrome-*f* reduction (~6 ms half-life) began ~1 ms post-flash. In ΔN, the apparent *b*-haeme reduction was enhanced because their oxidation slowed ~20-fold (Fig. 4b). This coincided with a ~10-fold slowdown in cytochrome-*f* reduction, indicating Q_i_ site impairment affecting the Q_o_ site as has been shown in *b*_6_ *f*^3,35^ and the related cytochrome *bc*_1_ complex^36,37^. Under anoxic conditions (Fig. 4c), the WT displayed flat *b*-haeme signals in the first 10 ms, followed by accelerated oxidation, consistent with prior findings^3,35^. In ΔN, a redox-inactive low-potential chain caused a ~25-fold slowdown in the high-potential chain, as reflected in cytochrome-*f* reduction (Fig. 4c,d).

The PetD N terminus we investigated is exposed to the aqueous phase, contains two lysine residues and is located near PetD Asp35. The latter is suggested to donate a proton to a water molecule in the Q_i_ site, where water displacement upon PQ binding has also been proposed^38^. It is possible that the ΔN truncation subtly affects the environment of the water channel or the functional properties of PetD Asp35. These changes may impair electron exchange in the low-potential chain, which could explain the pronounced slowdown in *b*_6_ *f* turnover at both Q_i_ and Q_o_ sites (Fig. 4 and Extended Data Fig. 8a). This slowdown is consistent with the diminished ETR, enhanced P700 donor-side limitation (Fig. 3) and growth retardation observed in ΔN (Extended Data Fig. 3a). Furthermore, diminished PQH_2_ oxidation at the Q_o_ site impedes STT7 activation^39^, which may account for the partial inactivation of the kinase in the ΔN mutant (Fig. 2a).

In contrast to ΔN, the phosphomimic T4E substitution does not affect *b*_6_ *f* function. However, both alterations interfere with STT7 function, albeit through distinct and probably partial mechanisms: Q_o_ site defect in ΔN and negative charge introduction in the PetD stromal part in T4E.

To better understand the basis of STT7 inactivation, we analysed its autophosphorylation pattern. Riche et al.^18^ demonstrated via PhosTag PAGE that multiple STT7 autophosphorylation events are required for kinase activation and that this process depends on the binding of STT7 to *b*_6_ *f*. Phosphorylation at residues Ser569 and Thr571 was strongly reduced in both ΔN and T4E strains, whereas other autophosphorylation sites, including Thr511/Thr513/Thr516, Ser533 and Thr547, were unaffected (Extended Data Fig. 5d–h). These data suggest that STT7 remains associated with the *b*_6_ *f* complex in both mutant backgrounds but fails to undergo full activation. The partial autophosphorylation pattern lacking residues Ser569 and Thr571 probably contributes to the reduced kinase activity observed. However, we cannot exclude the possibility that STT7 binding to *b*_6_ *f* is impaired in the two mutants.

Our findings point to a feedback mechanism in which STT7 activity is modulated by PetD Thr4 phosphorylation (Extended Data Fig. 1). This feedback mechanism complements previously described STT7 autophosphorylation pathways involving other stroma-exposed structures of the *b*_6_ *f* complex^17,18^. In some cases, such as PSBR, T4A mutants displayed elevated phosphorylation levels, consistent with disrupted feedback regulation. Two models may explain this phenomenon. STT7 bound to *b*_6_ *f* may detect PetD Thr4 phosphorylation and, in response, reduce its own autophosphorylation, leading to decreased activity. Alternatively, STT7-dependent phosphorylation of PetD Thr4 could hinder subsequent STT7 binding to the *b*_6_ *f* complex, thus indirectly limiting further kinase activation.

**Extended Data Fig. 9:**
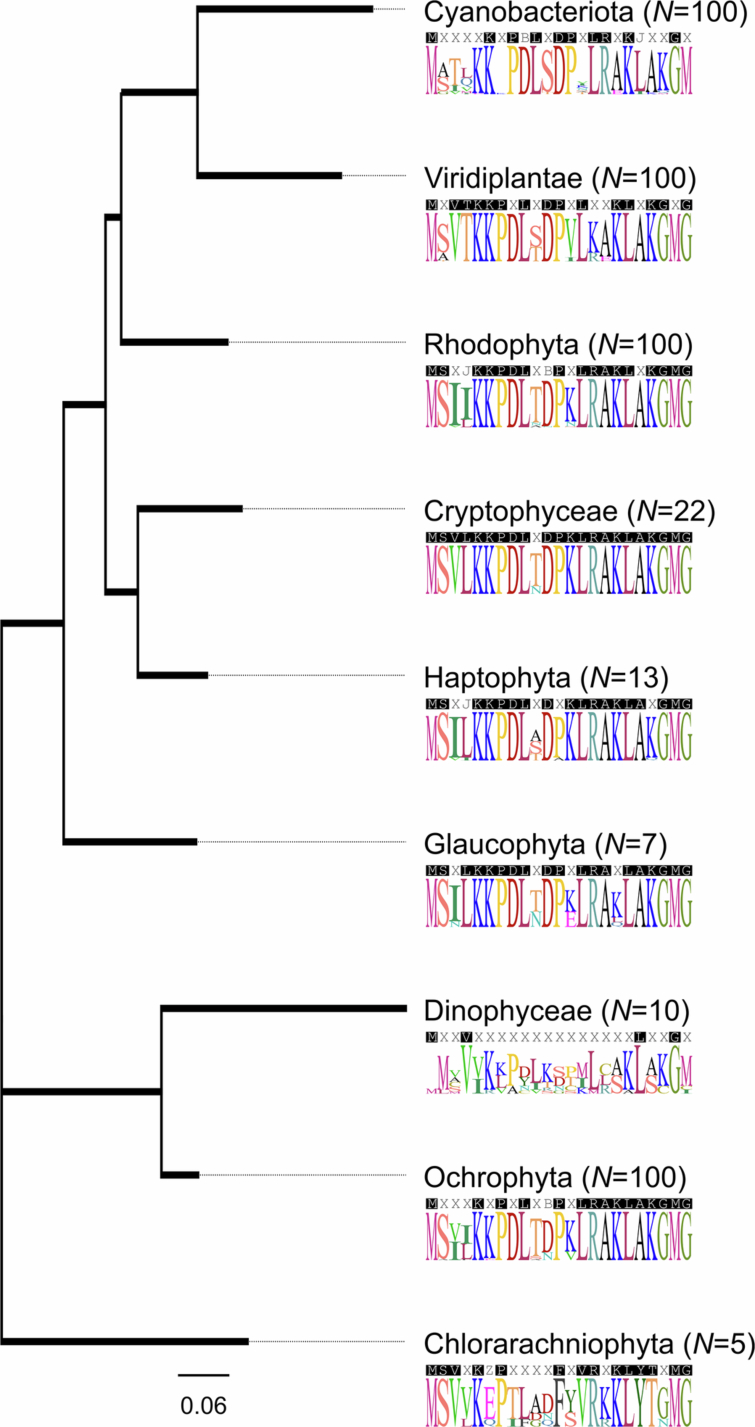
Alignments and phylogenetic tree of PetD highlights T4 in the green lineage. The consensus sequences were used for tree generation and were based on the first 100 BlastP hits (https://blast.ncbi.nlm.nih.gov/) for the *C. reinhardtii* PetD amino acid sequence. Hits were filtered for a Max Score above 100, followed removal of duplicate annotated sequences. The first 23 amino acids are shown.

We have shown that the N-terminal region of PetD is critical for *b*_6_ *f* function but not for complex stability, and that phosphorylation at PetD Thr4 regulates STT7 kinase activity via an autoregulatory feedback loop. This regulatory mode appears absent in organisms such as cyanobacteria^40^ and diatoms^41^, which lack PetD Thr4 and in which state transitions are differently realized (Extended Data Fig. 9). In Viridiplantae, feedback suppression of STT7 via PetD Thr4 may help coordinate state transitions with alternative regulatory mechanisms, including those targeting PSII core subunits such as PSBD Thr2 (refs. ^42,43^).

## Methods

### WT plasmid construction

The *petD*/*trnR1* region (481 bp upstream and 1,032 bp downstream of *petD*) was amplified from WT chloroplast DNA using restriction-site-containing primers 1 (SalI) and 2 (EcoRI; for the oligonucleotide sequences, see Supplementary Table 1). The SalI/EcoRI digested PCR product was ligated into a double-digested pUC18 cloning vector and propagated as pUC18-3′-*petD*-5′ in NEB 5-alpha *Escherichia coli* cells using ampicillin selection. In the next step, the aminoglycoside adenyl transferase (aadA) spectinomycin resistance cassette^44^ was amplified from a pUC18-aadA vector using primers 3 and 4, each containing a SpeI restriction site. The cloned *petD*/*trnR1* region contains a single SpeI site into which the aadA cassette was cloned. To do so, intermediate vectors were created. First (pUC18-int1), the SalI/SpeI fragment from pUC18-3′-*petD*-5′ (1.46 kb) was ligated with the SpeI-digested aadA PCR fragment, followed by PCR (primers 1 and 5) and blunt ligation of the ~3-kb product into a SmaI-digested empty pUC18 vector. Second (pUC18-int2), the EcoRI/SpeI fragment from pUC18-3′-*petD*-5′ (0.51 kb) was ligated with the SpeI-digested aadA PCR fragment, followed by PCR (primers 2 and 6), XmaI/EcoRI digestion of the ~2-kb product and ligation into an empty pUC18 vector. The 2.4-kb fragment of BamHI/BlpI digested pUC18-int1 was ligated with the 3.7-kb fragment of BamHI/BlpI-digested pUC18-int2 to yield pUC18-3′-*petD*-aadA-5′

### Point mutation and truncation plasmid construction

To generate T4E and T4A substitutions as well as the *petD* truncation ∆N (lacking the underlined N-terminal amino acids MSVTKKPD…), primers 8–11 and 13 were phosphorylated. Using pUC18-3′-*petD*-aadA-5′ as a template, five independent PCRs were done with the following primers: 1 and 8 (I), 1 and 9 (II), 1 and 10 (III), 11 and 12 (IV) and 12 and 13 (V). Ligated PCR fragments I and IV served as templates for the T4E PCR (primers 1 and 12), which was further digested (PacI/SalI) for ~1.36-kb insert swapping and mutant introduction into pUC18-3′-*petD*-aadA-5′ (ligation with a ~4.78-kb backbone). In a similar fashion, for T4A PCR (primers 1 and 12), PCR fragments II and IV served as templates. For ∆N PCR (primers 1 and 12), fragments III and V served as templates.

### Biolistic transformation of the WT strain

Mutant strains were generated through chloroplast transformation using plasmids containing the modified *petD* gene version along with an aadA cassette to confer spectinomycin resistance (150 µg ml^−1^). The recipient WT strain carried a six-histidine tag at the C terminus of PetA^21^. Spectinomycin-resistant clones were selected and replated several times to obtain homoplastic strains (for the sequencing primers, see Supplementary Table 1).

### Growth conditions and treatments

Cells were maintained at 50 µmol photons m^−2^ s^−1^ at 23 °C on agar plates of Tris–acetate–phosphate (TAP) medium or Tris-minimal medium (TP) in the absence of acetate^45^. The strains were checked for photoautotrophic growth for two weeks by spotting 20 µl of 3 × 10^5^ cells per ml (and dilutions) on TP agar plates at 25 °C kept under continuous light at 40 and 200 µmol photons m^−2^ s^−1^. All other functional measurements used synchronized cells under a 16-h light/8-h dark regime. Liquid TP culture growth was supported by bubbling sterile air. For state transition treatment using 77 K fluorescence, TAP-grown cultures were freshly diluted one day before the experiment and, upon harvesting (4,000 *g*, 5 min, 23 °C), adjusted to 5 µg chlorophyll ml^−1^. Aliquots were either kept for 45 min in aerobic conditions by shaking in 40 µmol photons m^−2^ s^−1^ light upon adding 10 µM DCMU (State 1) or given anoxic treatment in the dark using a glucose oxidase/catalase cocktail (State 2)^3^. For state transition time course experiments, TAP-grown cells (40 µg chlorophyll ml^−1^) were bubbled with argon gas for 15 min in the dark, followed by 1:1 dilution in TP-Ficol (20% w/v) and the addition of 10 µM DCMU. For proteomic analyses of *stt7-17* experiments, TAP-grown cells were shifted to State 2 conditions for 30 min, and samples for phosphoproteomic analyses were collected.

### Biochemical methods

Liquid cultures of the WT, T4A, T4E and ΔN were grown in TAP medium and freshly diluted one day before harvesting. The cells were pelleted and mixed in a buffer containing 0.2 M dichloro-diphenyl-trichloroethane, 0.2 M Na_2_CO_3_, 10 mM NaF and protease inhibitors (0.2 mM phenylmethylsulfonyl fluoride, 1 mM benzamidine and 20 mM amino caproic acid) to be analysed via SDS–PAGE^46^. For haeme staining, *b*_6_ *f* was isolated using the standard protocol^47^ to analyse its purity via SDS–PAGE. The high-spin haemes were stained in gel using 3,3′,5,5′-tetramethylbenzidine^48^. Blue Native PAGE was carried out according to the manufacturer’s protocol (NativePAGE Bis-Tris; Thermo Fisher Scientific).

### Generation of the *stt7-17* mutant with CRISPR–CAS9

*Chlamydomonas* strain CC-125 (*nit1*;*nit2*;*mt*^+^) was grown in TAP media under continuous illumination of 50 μmol photons m^−2^ s^−1^ at 22 °C. Single guide RNAs (sgRNAs) were designed by CHOPCHOP using v.5.6 of the *C. reinhardtii* genome. 5′-CTCCTGTAGACCGCTCAATGCGG was selected as the targeting sequence, and sgRNAs were bought with all accessory parts to make it functional (IDT). Ribonucleic protein complexes were prepared by duplexing the sgRNA (23% v/v; IDT) and Cas-9 (19% v/v; IDT) in a duplex buffer (58% v/v; IDT) and incubated at 37 °C for 25 to 30 min. After treatment with autolysin for 3 h without shaking, the cells were washed with TAP media supplemented with 40 mM sucrose, resuspended with the same supplemented media to approximately 2 × 10^8^ cells per ml and electroporated in the presence of a hygromycin resistance DNA cassette (2 µg ml^−1^) and the 37 °C-incubated ribonucleic protein mixture. After 10 min at room temperature, the cells were resuspended in 10 ml of TAP media supplemented with 40 mM sucrose and left without shaking overnight in dim light (10–20 µmol photons m^−2^ s^−1^) at 22 °C. Hygromycin-resistant transformants were selected on solid TAP media (2% agar w/v) containing hygromycin (20 µg ml^−1^). sgRNA-targeted regions were checked via PCR, using oligonucleotides STT7F/STT7R, and the gene lesion was characterized using Oxford Nanopore Technologies long-read sequencing (Extended Data Fig. 6 and Supplementary Table 1).

### Sample preparation for mass spectrometry

Protein isolation from cell pellets was carried out as described previously^43^. To disrupt the cells and extract proteins, lysis buffer (100 mM Tris-HCl (pH 8.5), 1 mM benzamidine, 1 mM PMSF, 10 mM sodium fluoride, 1 mM sodium orthovanadate, 10 mM sodium pyrophosphate, 10 mM β-glycerophosphate and 2% (w/v) SDS) was added to the cell pellets, and the samples were incubated for 15 min at 65 °C in a Thermomixer at 1,000 r.p.m., followed by centrifugation at 20,000 *g* for 10 min to pellet cell debris. Protein concentration in the supernatant was determined by BCA assay (Pierce BCA Protein Assay Kit, Thermo Fisher Scientific). A lysate volume corresponding to 100 µg of protein was tryptically digested according to a modified filter-aided sample preparation protocol^49^. Briefly, cell lysates were transferred to centrifugal ultrafiltration devices (Amicon Ultra, 0.5 ml, 30-kDa cut-off, Merck Millipore) and concentrated to 20 µl by centrifugation for 20 min at 14,000 *g* at room temperature. Reduction/alkylation of cysteines and tryptic digestion of proteins (100 µg per sample) were performed following the SP4 protocol^50^. After digestion, peptides were desalted using PurePep H50 SPE Spin columns (Affinisep). The peptide samples were divided into two aliquots: 5 µg for whole-proteome analysis and 95 µg for phosphopeptide enrichment. Both aliquots were dried using vacuum centrifugation before further processing.

To enrich phosphopeptides, titanium dioxide (TiO_2_) beads with 5-µm particle size (Titansphere, GL Sciences) in a metal oxide affinity chromatography procedure were used. TiO_2_ (1 mg per sample) was activated once with acetonitrile and equilibrated three times with loading buffer (80% (v/v) acetonitrile, 5% (v/v) TFA and 1 M glycolic acid). Loading buffer was added to create a 10% (w/v) TiO_2_ suspension. Peptide samples were dissolved in 50 µl of loading buffer, combined with 10 µl of TiO_2_ suspension and incubated for 30 min at 25 °C in an Eppendorf Thermomixer at 1,200 r.p.m. The mixture was transferred to SDB-XC STAGE tips prepared in-house^51^. Unless stated otherwise, all subsequent washing and elution steps were performed using 60-µl buffer volumes with in-between centrifugation at 2,000 *g* at room temperature. The buffer was removed by centrifugation, and the TiO_2_ beads were washed twice with loading buffer and once with 1% (v/v) acetonitrile and 0.1% (v/v) TFA (W1). Non-phosphorylated peptides that were removed from the beads and subsequently bound to the SDB-XC membrane plug were removed by one wash with 80% (v/v) acetonitrile and 0.1% (v/v) TFA (W2). The phosphopeptides were eluted from the TiO_2_ beads onto the SDB-XC material using 160 µl of pH 11 buffer (0.4 M sodium hydrogen phosphate and NaOH with 1% acetonitrile). After being washed once with 1% acetonitrile and 0.1% TFA, the phosphopeptides were sequentially eluted in three fractions using increasing concentrations of acetonitrile (7.5%, 20% and 60%) in 100 mM ammonium formate (pH 10). For samples from the *stt7-17* experiment, only one fraction (60% acetonitrile in 100 mM ammonium formate (pH 10)) was collected. The strongly bound phosphopeptides were eluted in two steps: first from the TiO_2_ beads using 5% ammonia and then from the SDB-CX membrane using 60% acetonitrile in 100 mM ammonium formate at pH 10. All fractions were dried by vacuum centrifugation and stored at −80 °C until mass spectrometry (MS) analysis.

### Mass spectrometry

#### Whole-proteome analysis

Dried peptide samples were reconstituted in 5 µl of 2% (v/v) acetonitrile and 0.05% (v/v) TFA in liquid chromatography (LC)/MS-grade water. The samples were analysed on an LC–MS/MS system consisting of an Ultimate 3000 NanoLC (Thermo Fisher Scientific) coupled via a Nanospray Flex ion source (Thermo Fisher Scientific) to a Q Exactive Plus mass spectrometer (Thermo Fisher Scientific). Peptides were concentrated on a trap column (Acclaim Pepmap C18, 5 mm × 0.3 mm, 3-µm particle size, Thermo Scientific) for 3 min using 4% (v/v) acetonitrile and 0.05% (v/v) TFA in LC/MS-grade water at a flow rate of 10 µl min^−1^. The trap column was operated in back-flush mode, allowing the transfer of peptides on a reversed-phase column (PepSep Fifty, 500 mm × 0.075 mm, 1.9-µm particle size, Bruker) for chromatographic separation. The eluents used were 0.1% (v/v) formic acid in LC/MS-grade water (A) and 0.1% (v/v) formic acid with 80% (v/v) acetonitrile in LC/MS-grade water (B). The gradient was programmed as follows: 2.5% to 5% B over 5 min, 5% to 17.5% B over 47 min, 17.5% to 40% B over 105 min, 40% to 99% B over 10 min and 99% B for 20 min. The flow rate was 250 nl min^−1^. The mass spectrometer was operated in data-dependent acquisition mode, alternating between one MS1 full scan and up to 12 MS2 scans. Full scans were acquired with the following settings: AGC target 3 × 10^6^, MS1 resolution 70,000, maximum injection time 50 ms and scan range 350–14,00 m/z. The settings for MS were AGC target 5 × 10^4^, resolution 17,500, MS2 maximum injection time 80 ms, intensity threshold 1 × 10^4^ and normalized collision energy 27 (HCD). Dynamic exclusion was set to ‘auto’, assuming a chromatographic peak width (FWHM) of 60 s.

#### Phosphoproteomic analysis

Prior to analysis, dried peptide samples were reconstituted in 5 µl of 2% (v/v) acetonitrile and 0.05% (v/v) TFA in LC/MS-grade water. The LC–MS/MS system, flow rate and eluent compositions were the same as described above. The gradient for peptide separation was as follows: 2.5% to 5% B over 5 min, 5% to 40% B over 92 min, 40% to 99% B over 10 min and 99% B for 20 min.

The mass spectrometer used the same data-dependent acquisition mode settings as in the whole-proteome analysis, with two changes: the MS2 maximum injection time was increased to 120 ms, and dynamic exclusion was set to 45 s.

### MS data analysis

For quantitative phosphoproteome analysis of four biological replicates, raw MS data from non-enriched and enriched samples were searched using Fragpipe^52,53^ against a concatenated, non-redundant database containing *C. reinhardtii* protein sequences from the v.5.6 and v.6.1 gene models (Phytozome 13, phytozome-next.jgi.doe.gov). In addition, the polypeptide sequences of mutant/tagged proteins were added to the database. The default ‘LFQ-MBR’ workflow settings were applied to data from whole-proteome samples, while the ‘LFQ-phospho’ workflow was used for phosphopeptide samples. A false discovery rate of 1% was applied at both peptide and protein levels.

Fragpipe output files containing peptide/protein identifications and quantitative data (‘combined_modified_peptide.tsv’ from enriched samples and ‘combined_protein.tsv’ from whole-proteome samples) were reformatted using a custom Python script (for code, see Supplementary Table 1) to ensure compatibility with Phospho-Analyst (v.1.0.2), which was used for differential expression analysis^54^: Missing values were imputed using the MinProb function, and variance stabilizing transformation was applied to normalize phosphosite intensities. Since the whole-proteome LFQ data were already normalized in Fragpipe, no additional normalization steps were needed. Phospho-Analyst automatically corrected phosphosite abundance changes for underlying protein levels. A phosphorylation site localization probability threshold of 0.33 was applied, and *P* values were adjusted using the Benjamini–Hochberg method. Predicted protein localizations are based on recently published data (PB-Chlamy)^55^.

### Optical spectroscopy

The flash-induced *b*_6_ *f* measurements were carried out by using a Joliot-type spectrophotometer (JTS-150, SpectroLogiX) equipped with the interference filters and deconvolution procedures as reported previously^3^. Cells were adapted to alternating 7.5 s darkness and 5 s AL (550 µmol photons m^−2^ s^−1^ peaking at 630 nm). Sub-saturating 6-ns flashes (Q-switched Nd:YAG, Continuum) were fired at 3 s darkness, hitting ~40% of PSI. Three averaged baseline spectra (in the absence of a flash) were subtracted from each flash measurement at the *b*_6_ *f*-specific wavelengths. For multiple turnover conditions, the previously described conditions and protocols used cells that were adapted to alternating 2.5 s AL and dark. Briefly, ETR were obtained in the presence of linear electron flow but referred to PSI charge separation activity. This stems from separate ECS calibration measurements that allowed, via saturating single-turnover flashes, for the conversion of optical changes (obtained as Δ*I*/*I* at 520 nm–546 nm) into the ECS signals corresponding to one PSI charge separation^56^. The ETR protocol was based on a dark-interval relaxation kinetics approach^57,58^. P700 measurements relied on the method of Klughammer and Schreiber^59^. State transition kinetics were measured by two independent methods using the JTS-150, starting with cells in State 2. AL (under the same conditions as above) was applied to induce the transition to State 1 in the presence of 10 µM DCMU. First, maximal chlorophyll fluorescence of dark-adapted (*F*_m_) and light-adapted (*F*_m_′) samples was recorded using saturating light pulses (250 ms, 7,000 µmol photons m^−2^ s^−1^). An increase in *F*_m_′ indicates an enlargement of the PSII antenna cross-section during the treatment, with the first measurement taken after 10 s of AL + DCMU illumination. Second, under the same conditions and as described elsewhere^26^, PSI-driven charge separation rates were determined from linear ECS slopes measured during 5-ms light pulses. The kinetics of ECS slopes reflect the decrease in PSI antenna size during the AL + DCMU treatment.

### Reporting summary

Further information on research design is available in the Nature Portfolio Reporting Summary linked to this article.

## Supplementary information

Reporting Summary

Supplementary Table 1 Phosphorylation data, protein fold changes, primers for the *stt7-17* and *petD* mutant backgrounds, and the genomic DNA sequence for *stt7-17*.

## Source data

Source Data Fig. 1 Statistical source data from spectroscopy.

Source Data Fig. 3 Statistical source data from spectroscopy.

Source Data Fig. 4 Statistical source data from spectroscopy.

Source Data Extended Data Fig. 7 Statistical source data.

Source Data Extended Data Fig. 8 Statistical source data.

## Data Availability

All data supporting the findings of this study are available within the paper and the Extended Data figures. The mass spectrometry proteomics data have been deposited to the ProteomeXchange Consortium (proteomecentral.proteomexchange.org) via the PRIDE partner repository^60^ with the dataset identifier PXD060640. The generated mutant cell lines are available on request from the authors. The *stt7-17* mutant is available via the Chlamydomonas Resource Center (CC-6272; www.chlamycollection.org). Source data are provided with this paper.
